# Super-Hydrophobic Macroporous TPU Gels for Oil–Water Separation and Organic Pollutant Adsorption

**DOI:** 10.3390/gels12100866

**Published:** 2026-09-24

**Authors:** Jingjing An, Xianbo Zhou, Xuan Zhou, Yingying Wang, Zhihao Lin, Feng Xu, Zhenxing Fang

**Affiliations:** 1 Ningbo Key Laboratory of Agricultural Germplasm Resources Mining and Environmental Regulation, College of Science and Technology, Ningbo University, Ningbo 315300, China; anjingjing@nbu.edu.cn (J.A.);; 2 School of Chemical Engineering and Life Health, NingboTech University, No. 1 South Qianhu Road, Ningbo 315100, China; zxb@nit.zju.edu.cn

**Keywords:** macroporous TPU gel, superhydrophobicity, oil–water separation, emulsion adsorption

## Abstract

Marine oil spills and plastic waste accumulation represent two critical environmental threats that urgently require integrated remediation strategies. In this work, we address both issues simultaneously by developing a facile, template-free, water-assisted thermally induced phase separation approach to fabricate superhydrophobic macroporous thermoplastic polyurethane (TPU) gels from recycled TPU pellets. Under optimal conditions (8% TPU, 9:1 1,4-dioxane/water, 0 °C, 30 min), the gel exhibits an interconnected macroporous network with ~84% porosity and a BET surface area of 7.67 m^2^/g. Notably, it achieves intrinsic superhydrophobicity (150.3°) without any additives or surface modifications. The gel shows high absorption capacities (5.39–55.64 g/g), rapid kinetics (equilibrium within 15 s), and excellent recovery efficiency (>97%) in pump-assisted separation. Beyond oil/water mixtures, we systematically investigate trace organic pollutant adsorption from oil-in-water emulsions, achieving up to 97% removal efficiency. This work establishes a closed-loop “waste-to-resource” strategy, positioning recycled-TPU-derived macroporous gels as cost-effective candidates for oil spill remediation and wastewater treatment.

## 1. Introduction

In recent years, marine crude oil spills—such as the notorious Deepwater Horizon incident—have become increasingly prevalent, releasing vast quantities of petroleum into the ocean and causing severe ecological damage while wasting valuable non-renewable oil resources. Meanwhile, oily organic wastewater accounts for a substantial proportion of the industrial effluents discharged by chemical and pharmaceutical plants, posing significant threats to both aquatic ecosystems and public health [1,2,3,4,5,6]. Conventional treatment technologies for oily wastewater, including chemical, biological, and physical methods, are typically hindered by long processing times, low separation efficiency, poor recyclability, and a propensity for secondary pollution. In contrast, adsorption materials such as hydrophobic porous materials [7,8,9,10,11,12], polymer aerogels [13,14,15,16,17,18], and so on [19,20,21], have attracted considerable research interest due to their excellent oil–water selectivity and high adsorption efficiency.

Thermoplastic polyurethane (TPU), benefiting from its excellent stability, chemical resistance, resilience, and mechanical properties, is extensively employed in packaging, sound insulation, filtration media, and structural materials for construction and automotive applications [22,23,24]. However, its massive usage has generated vast quantities of plastic waste. Owing to its recalcitrant nature, such waste causes considerable damage to soil and water environments. Conventional disposal routes, such as landfilling and incineration, inevitably lead to secondary contamination while also wasting valuable plastic resources. Given its intrinsic nature, TPU theoretically possesses the potential to adsorb oily organic pollutants. Therefore, its application in oil–water separation or organic contaminant removal offers a promising strategy to achieve the goal of “using waste to treat waste”.

Among various fabrication approaches, thermally induced phase separation (TIPS) has been widely employed to prepare porous polymeric foams for oil–water separation [25,26,27,28,29,30]. For instance, porous TPU foams obtained via TIPS have demonstrated efficient absorbability toward various oils and organic solvents, owing to their intrinsic hydrophobicity [31,32,33]. However, these materials have generally fallen short of superhydrophobicity. Furthermore, systematic investigations regarding the influence of key fabrication parameters—such as phase separation time and temperature—remain lacking, and the adsorption performance toward trace organic pollutants in oil-in-water emulsions has yet to be explored.

Despite these progresses, several critical aspects remain largely unexplored: the use of recycled TPU as a feedstock, the achievement of intrinsic superhydrophobicity without any additive or surface modification, the adsorption behavior toward trace organic pollutants in oil-in-water emulsions, and the validation of continuous separation performance under pump-assisted conditions. To the best of our knowledge, this integrated strategy—combining recycled feedstock, additive-free superhydrophobicity, emulsion-phase pollutant adsorption, and engineering-scale separation validation—has not been previously reported for TPU-based macroporous gels.

In this work, we address these gaps by developing a facile water-assisted thermally induced phase separation method combined with freeze-drying to fabricate macroporous TPU gels from recycled TPU pellets. Unlike the vast majority of previous studies that rely on virgin polymers, nanoparticle incorporation, or surface chemical modification [10,11,12,31,32,33], our approach offers a closed-loop “waste-to-resource” strategy that simultaneously tackles plastic waste accumulation and oil spill remediation. The effects of water dosage, TPU concentration, phase separation time, and phase separation temperature on the morphology, hydrophobicity, and absorbability of the resulting gels were systematically investigated. In addition, the adsorption performance toward trace organic pollutants in oil-in-water emulsions was systematically evaluated, and the continuous separation capability was validated through pump-assisted experiments. The combination of high porosity (~84%), interconnected macroporous network, and superhydrophobicity positions this material as a cost-effective and scalable adsorbent for oil spill remediation and organic pollutant removal.

## 2. Results and Discussion

### 2.1. Morphology of the Macroporous Tpu Gels

The morphologies of macroporous TPU gels prepared with different TPU concentrations were observed by SEM at different magnifications. As shown in Figure 1a, Sample I-1 (5% TPU) exhibits an irregular porous morphology; although some interconnectivity between pores is observed, the pore size distribution is non-uniform. In contrast, sample I-2 (8% TPU) displays a three-dimensionally interconnected porous structure with a narrower pore size distribution and less variation in skeleton dimensions, overall presenting a hollow, spherical porous architecture. Sample I-3 (11% TPU), however, is predominantly composed of closed cell structures, exhibiting poor interconnectivity and a honeycomb-like morphology. Notably, sample I-2 exhibits a novel and uniform hierarchical macroporous structure and a high porosity of 84%.

Figure 2 presents the morphology of macroporous TPU gels prepared with different 1,4-dioxane/water ratios. When the ratio was 15:1 (Figure 2a), no three-dimensionally interconnected structure was formed; the pore sizes were predominantly in the range of 50–150 μm, with relatively thick skeletal struts and intact pore walls. At a ratio of 9:1 (Figure 2b), a well-defined three-dimensionally interconnected porous morphology was obtained, with pore sizes predominantly around 80 μm and uniform skeleton dimensions. When the ratio was 6.3:1 (Figure 2c), the interconnected structure was again absent, and the pore size distribution became bimodal, featuring large pores of 100–200 μm alongside small pores of 10–30 μm, with the overall morphology tending toward discrete microsphere-like structures. This morphological transition from an interconnected porous framework to microspheres with increasing water content—from isolated macropores (15:1) to a fully interconnected network (9:1) and further to microsphere-dominated morphology (6.3:1)—can be attributed to the change in phase separation pathway. In this system, water acts as a non-solvent. At low water content, spinodal decomposition dominates, forming a bicontinuous structure; as water content increases beyond a critical threshold, nucleation and growth become predominant, leading to the formation of discrete droplets that solidify into microspheres upon freeze-drying. This is consistent with the classical non-solvent-induced phase separation (NIPS) mechanism, where higher non-solvent content favors droplet-type morphology [34].

At a phase separation time of 15 min (Figure 3a), the pore walls appeared relatively intact with few visible interconnecting openings, suggesting limited pore connectivity compared with the sample obtained at 30 min. When the time was extended to 30 min (Figure 3b), abundant interconnected pores were observed on the pore walls, with uniform skeletal and pore dimensions, collectively forming a three-dimensionally interconnected hollow spherical structure. Further prolonging the phase separation time to 45 min (Figure 3c), the pore walls appeared distorted and partially fused, with broken struts and loss of the regular hollow spherical architecture observed at 30 min. This morphological degradation—characterized by wall thickening, pore shrinkage, and structural irregularity—is indicative of skeleton collapse, likely resulting from prolonged coarsening and Ostwald ripening during the extended phase separation period.

At the same phase separation time, the sample prepared at 4 °C exhibited a non-uniform pore size distribution, a greater abundance of large pores, and inferior interconnectivity compared with that obtained at 0 °C (Figure 3d,e). This phenomenon is ascribed to the fact that at 4 °C, deionized water remains in the liquid state; during cooling, it tends to accumulate at the interface between the TPU and 1,4-dioxane phases, thereby promoting the formation of large pores.

In view of the above, a phase separation temperature of 0 °C and a phase separation time of 30 min are considered optimal for generating a well-defined, three-dimensionally interconnected microstructure with uniform pore size distribution and regular morphology.

Based on the systematic evaluation of all fabrication parameters discussed above—including TPU concentration, solvent ratio, phase separation temperature, and phase separation time—the optimal conditions were established as follows: 8% (*w*/*v*) TPU, 9:1 (*v*/*v*) 1,4-dioxane/water, 0 °C phase separation temperature, and 30 min phase separation time. The resulting material (sample I-2) exhibits a superior hierarchical porous structure with a regular pore morphology.

Nitrogen adsorption–desorption measurements were performed to further characterize the porous structure of sample I-2. The BET specific surface area was determined to be 7.67 m^2^/g, which is consistent with a macroporous-dominated architecture (pore sizes > 50 nm) and corroborates the SEM observations. Although the specific surface area is moderate, the ultra-high porosity (~84%) and the fully interconnected macroporous network provide ample void space and rapid transport pathways for oil absorption. Notably, this combination of high porosity and interconnected macroporosity was achieved without the use of any nanoparticles, templates, or surface chemical modifications, distinguishing our water-assisted TIPS approach from many reported strategies that rely on complex composite formulations or post-treatment modifications to achieve similar porous architectures [10,11,12,31,32,33].

### 2.2. Wettability of the Macroporous Tpu Gels

The hydrophobic and oleophilic properties of macroporous TPU gels were characterized by water and oil contact angle (CA) measurements. As shown in Figure 4a,b, water droplets (dyed with Methyl blue) maintained a stable spherical shape on the cross-section of sample I-2, yielding a water CA of 150°, indicating high hydrophobicity without any surface treatment. Figure 4c demonstrates the oil wettability: a droplet of vegetable oil (dyed with Sudan IV) was immediately and completely absorbed within 1 s upon contacting the sample surface, suggesting an oil CA of approximately 0° (Figure 4d). These observations confirm the superhydrophobicity and superoleophilicity nature of the macroporous TPU gel.

Figure 4e presents the water CA values of the TPU film and macroporous TPU gels prepared under various experimental parameters. All TPU gel samples exhibited hydrophobic behavior, with water CAs exceeding 120°, in contrast to the hydrophilic TPU film (water CA = 85.7°). Notably, Sample I-2 achieved the maximum water CA of 150.3°, which satisfies the criterion for superhydrophobicity (≥150°). This superhydrophobicity is primarily attributed to the three-dimensional macroporous architecture with a relatively uniform pore size distribution. Given its superior hydrophobicity and well-defined macroporous structure, Sample I-2 was selected for further investigation in the subsequent sections. A more comprehensive wettability analysis, including determination of the polar and dispersive components of surface energy using multiple probe liquids, would provide deeper insight into the wetting mechanisms and is planned for future investigations.

### 2.3. Oil Absorbability

Figure 5a illustrates the selective absorption ability of sample I-2 toward oil/organic solvent-water mixtures. Owing to its strong capillary effect, sample I-2 can rapidly and completely absorb vegetable oil within 13 s (A1–A4) or CCl_4_ within 3 s (B1–B3), regardless of whether the pollutant floats on or sinks in water.

The saturated absorption capacities (*Q_s_*) of Sample I-2 for various oils and organic solvents are presented in Figure 5b. All *Q_s_* values are in the range from 5.39 to 55.64 g/g, corresponding to 5.12–50.71 times the material’s own weight, depending on the viscosity and density of the absorbed liquids. These values are superior to those of many previously reported absorbents, which is attributed to the material’s superoleophilicity and high porosity providing sufficient storage space [35].

Figure 5c depicts the absorption kinetics of sample I-2 toward vegetable oil (chosen as a representative due to its high viscosity). The absorption equilibrium was reached within 15 s, indicating a rapid absorption rate. The absorption rate constant (*k*) derived from the pseudo-first-order kinetics model (inset of Figure 5c) is 0.383, with a high correlation coefficient (*R*^2^ = 0.955), confirming that the absorption kinetics conform to this model.

### 2.4. Reusability and Recyclability

Figure 6a illustrates a typical absorption-squeezing process using a hypodermic syringe to recover oil from water. The cyclic absorption capacities of sample I-2 for vegetable oil are shown in Figure 6b. It is worth noting that the *Q_s_* value increased slightly in the second cycle, likely because the high viscosity of the oil hinders complete removal by manual squeezing. Nevertheless, after ten cycles, the sample retained a stable *Q_s_* value of over 98.3% and could still absorb up to six times its own weight of vegetable oil.

To further evaluate its potential for large-scale oil spill remediation, a pump-assisted continuous absorption system was implemented [36]. As shown in Figure 6d, sample I-2 (0.225 g) was connected to a vacuum pump via a transparent rubber tube and immersed in a mixture of vegetable oil (dyed with Sudan IV) and water (dyed with Methylene blue). No water was drawn into the collected oil during the process, confirming effective oil–water separation. The separation efficiency remained stable at approximately 97%, and the system could continuously separate oil or organic solvents equivalent to over 91 times its own weight within ten cycles. These results underscore the practical potential of this pumping approach for mitigating large-scale crude oil spills.

Although the manual squeezing experiments (Figure 6a,b) have demonstrated the structural robustness of the material over ten cycles, a more comprehensive mechanical evaluation—including compression-recovery and stress–strain tests—would provide additional quantitative insights into its long-term durability. This is planned for future work.

### 2.5. Adsorption and Removal of Trace Organic Solvents from Oil-in-Water Emulsions

To evaluate the adsorption performance of sample I-2 toward trace toluene in the oil-in-water organic pollutant emulsion, the absorbance of the emulsion was measured by UV-Vis spectrophotometry at predetermined time intervals, enabling continuous monitoring of the toluene concentration variation. At a fixed wavelength of 260 nm, the residual concentration (*C_f_*) was determined based on the measured absorbance and the known initial concentration (*C_0_*), assuming a linear relationship between absorbance and concentration. The adsorption efficiency was subsequently calculated according to Equation (4). The detailed data are summarized in Table 1.

At a fixed initial concentration, the absorbance decreased progressively with increasing adsorption time, with the adsorption efficiency reaching 93.4% at 6 h for a toluene volume of 1 mL. A similar trend was observed at higher toluene concentrations. Furthermore, for a given adsorption time, a higher initial toluene concentration in the oil-in-water emulsion led to greater adsorption efficiency —reaching 97% at 6 h for a toluene volume of 2 mL. Temperature also exerted a significant influence: at 60 °C, the adsorption efficiency reached 88% within only 3 min for a toluene volume of 2 mL. This enhancement is attributed to the fact that elevated ambient temperatures intensify the molecular motion of organic pollutants, thereby facilitating their uptake by the macroporous TPU gel. However, it should be noted that excessive temperature is not necessarily advantageous, as the structural integrity of the polymeric porous adsorbent must be considered. For the TPU gel in particular, overheating may cause softening and collapse of the porous skeleton, leading to structural degradation and consequent loss of adsorption performance.

## 3. Conclusions

In summary, superhydrophobic macroporous TPU gels were successfully fabricated via a simple water-assisted thermally induced phase separation method combined with freeze-drying. Under optimized conditions (8% TPU, 9:1 1,4-dioxane/water, 0 °C phase separation temperature, and 30 min phase separation time), the resulting gel exhibits a highly interconnected three-dimensional macroporous network with uniform pore dimensions, a porosity of ~84%, and a BET specific surface area of 7.67 m^2^/g, confirming its predominantly macroporous character. Notably, the gel achieves intrinsic superhydrophobicity (water CA = 150.3°) without any nanoparticle incorporation, chemical coating, or surface modification, offering a simpler and more environmentally benign alternative to composite-based strategies. It demonstrates high saturated absorption capacity (5.39–55.64 g/g), rapid absorption kinetics (equilibrium within 15 s), and excellent oil recovery efficiency (>97%) in pump-assisted separation. Furthermore, it shows promising adsorption performance toward trace organic pollutants in oil-in-water emulsions. These features, combined with the use of recycled TPU as the raw material, position the developed macroporous gel as a cost-effective, environmentally friendly, and high-performance candidate for oil spill cleanup and organic pollutant removal, demonstrating a closed-loop “waste-to-resource” strategy.

While the current work successfully demonstrates the feasibility of converting recycled TPU into high-performance macroporous gels, further investigations into the long-term durability of the gel are warranted. These include systematic evaluation of its mechanical robustness (e.g., compression-recovery behavior), thermal stability retention during prolonged use, and structural evolution under repeated absorption-squeezing cycles. Such studies will provide a more comprehensive understanding of the material’s service life and practical reliability in oil spill remediation.

## 4. Materials and Methods

### 4.1. Materials

The recycled TPU pellets were prepared in-house from virgin TPU pellets (Elastollan 1185A, BASF Polyurethanes GmbH, Lemförde, Germany) by a multi-step process: twin-screw extrusion, granulation, pulverization, and re-granulation. These recycled pellets were then used as the raw material for macroporous gel fabrication. The recycled TPU exhibited decomposition temperatures (T_5%_ = 335.9 °C, T_50%_ = 412.8 °C), which are comparable to those of virgin TPU (T_5%_ = 334.3 °C, T_50%_ = 422.5 °C), which were performed on a STA 449 simultaneous thermal analyzer (NETZSCH-Gerätebau GmbH, Selb, Germany) and heated from 50 to 650 °C at a heating rate of 20 °C/min under a nitrogen atmosphere.

SDS (sodium dodecyl sulfate), 1,4-dioxane, chloroform, cyclohexane, carbon tetrachloride, ethanol, toluene and acetone were bought from Shanghai Aladdin Biochemical Technology Co., Ltd., Shanghai, China. Vegetable oil, silicone oil, distilled water, coloring agents (Methyl blue for water and Sudan Dyes IV for oil) were used as received.

### 4.2. Fabrication of Macroporous Tpu Gels

Recycled TPU pellets were completely dissolved in 1,4-dioxane under rapid stirring at 70 °C for 2 h to form a homogeneous solution. Under vigorous magnetic stirring, varying amounts of deionized water were added to the solution. Subsequently, the obtained solution was rapidly transferred into a flat-bottomed test tube, which was immediately placed in a water bath at the designated temperature for a predetermined time to induce phase separation and subsequent coarsening. The tube was then moved to a refrigerator at −24 °C and kept for 48 h to allow complete phase separation and freezing. Finally, the frozen sample was lyophilized in a freeze dryer (FreeZone 4.5 Liter Benchtop Freeze Dryer, Labconco Corporation, Kansas City, MO, USA) at −56 °C under a pressure of 10 Pa for 72 h, yielding the final macroporous TPU gels. For comparison, the TPU concentration (*w*/*v*), 1,4-dioxane/water ratio (*v*/*v*), phase separation time and temperature were varied, and the corresponding samples were labeled as listed in Table 2.

### 4.3. Characterization of the Macroporous Tpu Gels

#### 4.3.1. Surface Morphology

The morphology of the macroporous TPU gels was observed using a scanning electron microscope (SEM, Hitachi SU8020, Hitachi High-Technologies Corporation, Tokyo, Japan) with an acceleration voltage of 5 kV.

#### 4.3.2. Porosity

The porosity of samples was measured using the ethanol saturated method (ESM), which is defined as the percentage of the pore volume in the entire porous material. The porosity (*φ*) is calculated by the following formula [31]:

(1) $$\varphi =\frac{(W_{0}-W)\;\rho }{{\rho W}_{0}+(\rho -\rho_{0})\;W}\times 100\%$$

*W_0_* and *W* represent the mass of the sample after saturation and ethanol removal, respectively; *ρ* and *ρ_0_* are the density of TPU and ethanol, respectively.

Nitrogen adsorption–desorption measurements were carried out at 77 K using a surface area analyzer (ASAP 2020, Micromeritics Instrument Corporation, Norcross, GA, USA). The specific surface area was determined by the Brunauer–Emmett–Teller (BET) method.

#### 4.3.3. Wettability

Contact angle (CA) measurements were performed on a HARKE-SPCA instrument (Beijing HARKE Experimental Instrument Co., Ltd., Beijing, China) at ambient temperature. The droplet volume was 3.0 µL. Each sample was measured and averaged from at least three different points.

### 4.4. Oil Absorption Measurements

The oil/organic solvent absorption performance of the macroporous TPU gels was evaluated by immersing a pre-weighed gel sample in 60 mL of the test liquid for 5 min at ambient temperature. After complete absorption, the saturated sample was gently shaken to remove excess surface liquid and reweighed. The saturated absorption capacity (*Q_s_*) was then calculated using Equation (2) [32]:

(2) $$Q_{s}=\frac{m_{1}-m_{0}}{m_{0}}$$

where *m_1_* and *m_0_* are the weights of the gel before and after absorption, respectively.

Absorption kinetics were monitored by placing the TPU gel onto the surface of vegetable oil (chosen as a representative due to its high viscosity) and recording the weight increase over time until equilibrium was reached. The kinetic data were analyzed using the pseudo-first-order model [37,38] given by Equation (3):

(3) $$\ln\left(Q_{s}-Q_{t}\right)=\ln Q_{s}-kt$$

where *Q_t_* is the sorption capacity at time t, *t* represents the sorption time, and *k* is the absorption rate constant.

### 4.5. Adsorption Toward Trace Organic Pollutants in Oil-in-Water Emulsions

The oil-in-water emulsion containing trace toluene (1.6%, *v*/*v*) was prepared by dissolving 0.15 g of SDS in 60 mL of distilled water, followed by the addition of 1 mL of toluene and stirring for 2 h. The saturation adsorption capacity of the macroporous TPU gel for toluene was predetermined as TPU/toluene = 1/10.9 (g/g); accordingly, 0.08 g of TPU gel was calculated to be sufficient for complete adsorption of 1 mL of toluene. This amount of TPU gel was added to the emulsion and stirred on a magnetic stirrer, with absorbance measurements taken at 3 min, 2 h, and 6 h using UV-Vis spectrophotometry (Cary 300, Agilent Technologies, Inc., Santa Clara, CA, USA). A reference solution of 0.15 g SDS in 60 mL water was used as the blank. The absorbance of the toluene-in-water emulsion was recorded over 190–400 nm before and after adsorption. The removal efficiency (*R_e_*) was calculated using Equation (4) [32]:

(4) $$R_{e}=\frac{C_{0}-C_{f}}{C_{0}}\times 100\%$$

where *R_e_* is the removal efficiency, *C_0_* is the initial concentration, and *C_f_* is the residual concentration after a given adsorption time.

The residual concentration *C_f_* was determined from the measured absorbance based on the linear relationship between absorbance and concentration (Beer–Lambert law).

## Figures and Tables

**Figure 1:**
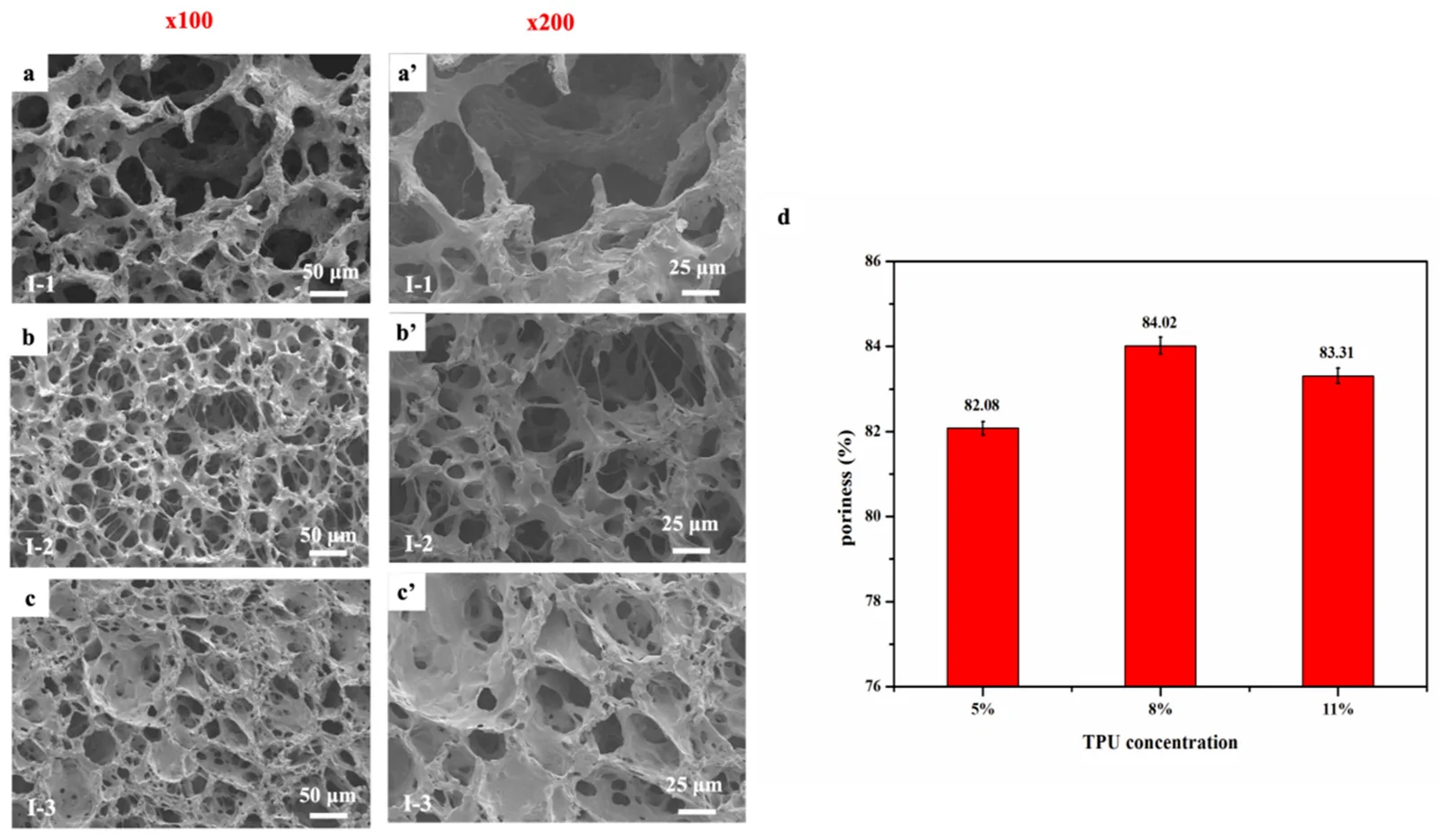
Morphologies ((**a**–**c**), ×100; (**a′**–**c′**), ×200) and Porosity (**d**) of macroporous TPU gels as a function of TPU concentration.

**Figure 2:**
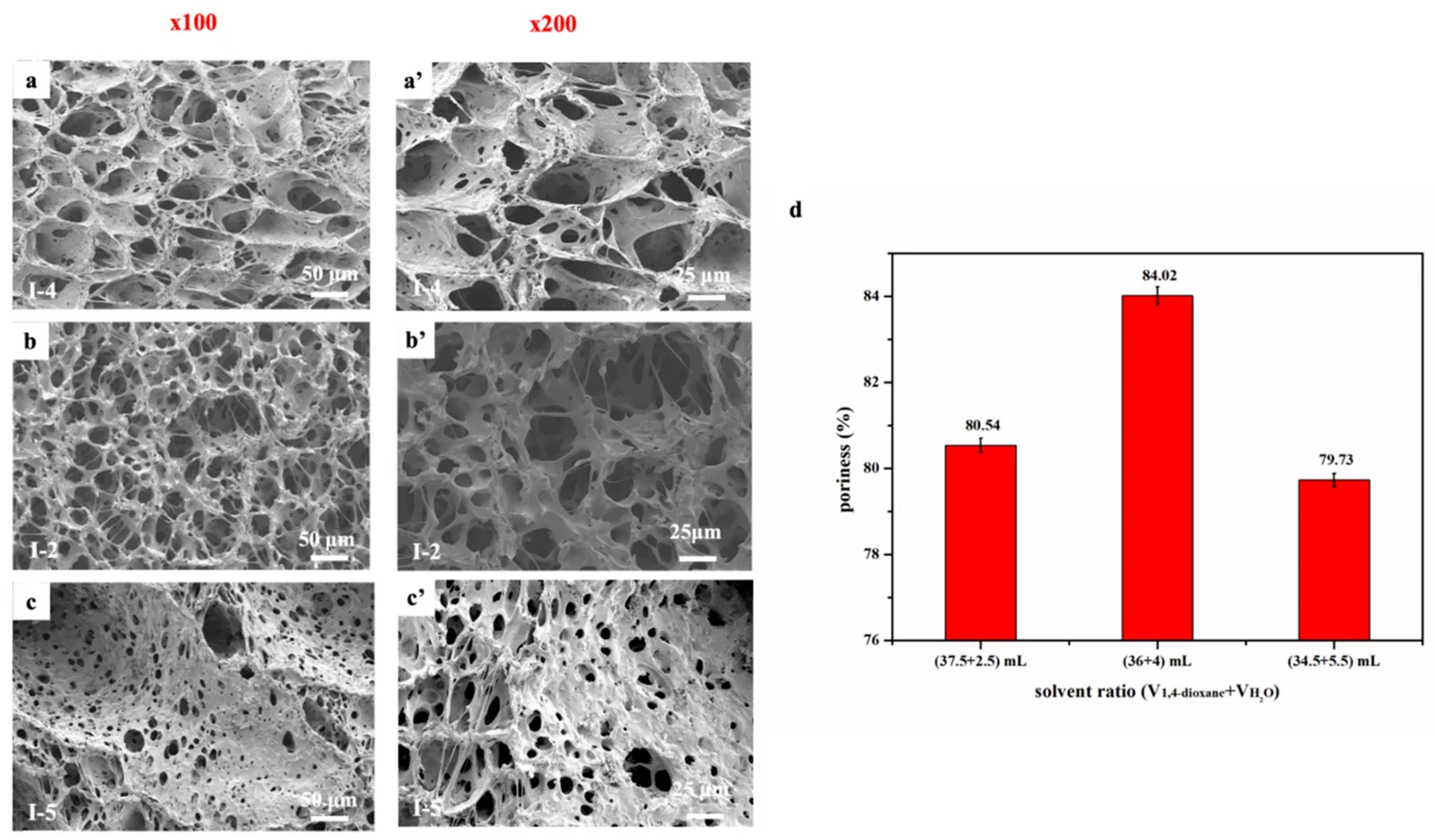
Morphologies ((**a**–**c**), ×100; (**a′**–**c′**), ×200) and Porosity (**d**) of macroporous TPU gels as a function of 1,4-dioxane/water (*v*/*v*).

**Figure 3:**
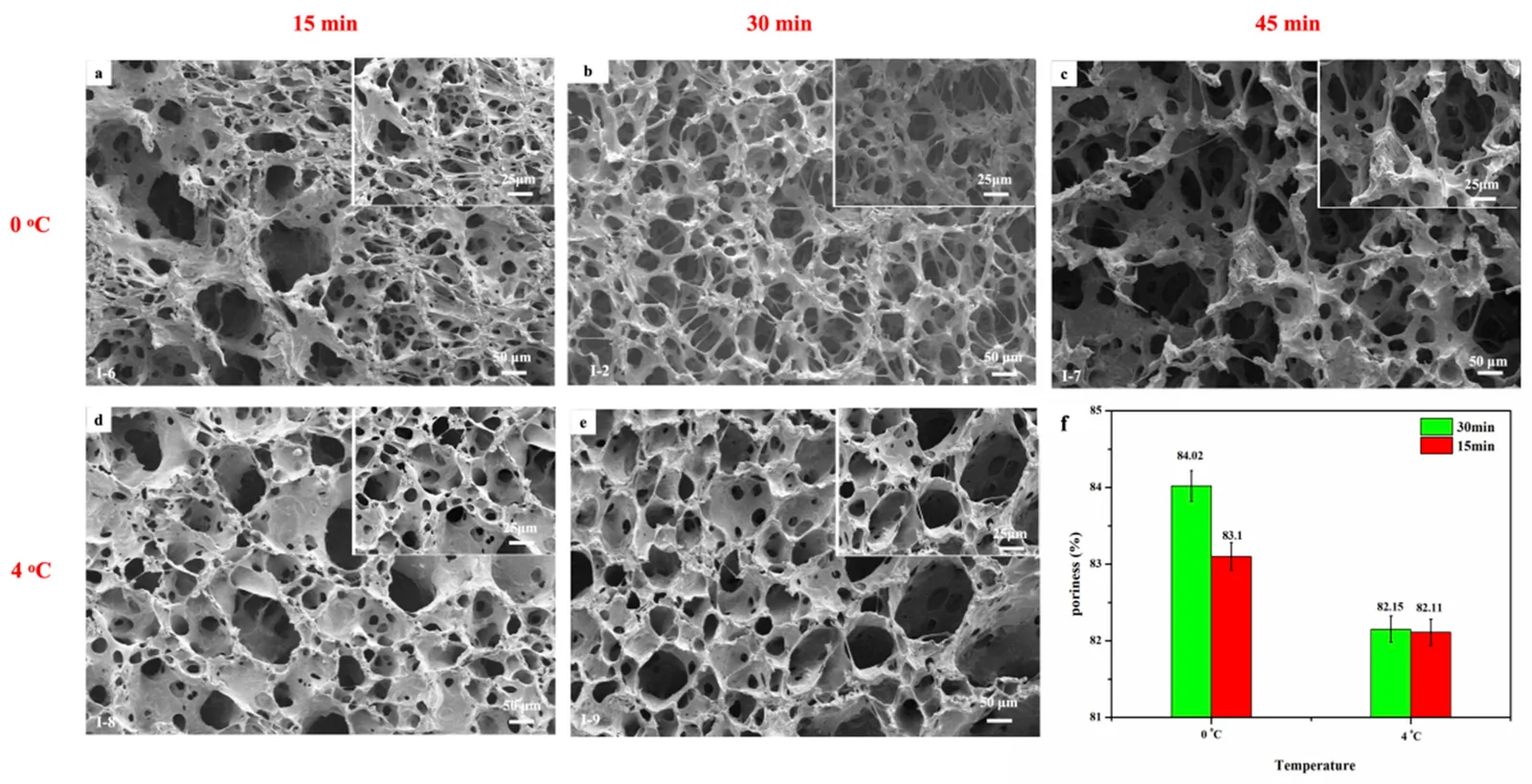
Morphologies (**a**–**e**) and Porosity (**f**) of macroporous TPU gels as a function of phase separation time and temperature.

**Figure 4:**
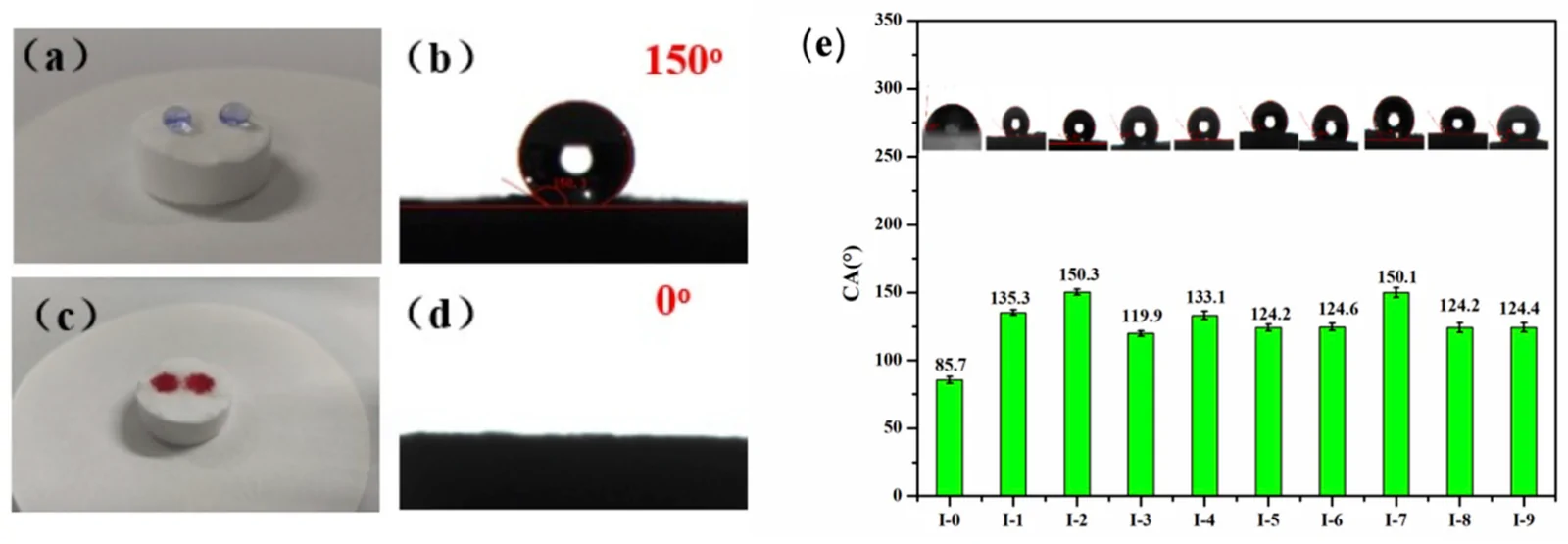
Photographs of (**a**) water droplets (dyed with methyl blue) and (**c**) vegetable oil (dyed with Sudan Dyes IV) on the cross section of sample I-2; (**b**) the CA value of water droplets; (**d**) the CA value of vegetable oil; (**e**) water CA values of the TPU film and macroporous TPU gels.

**Figure 5:**
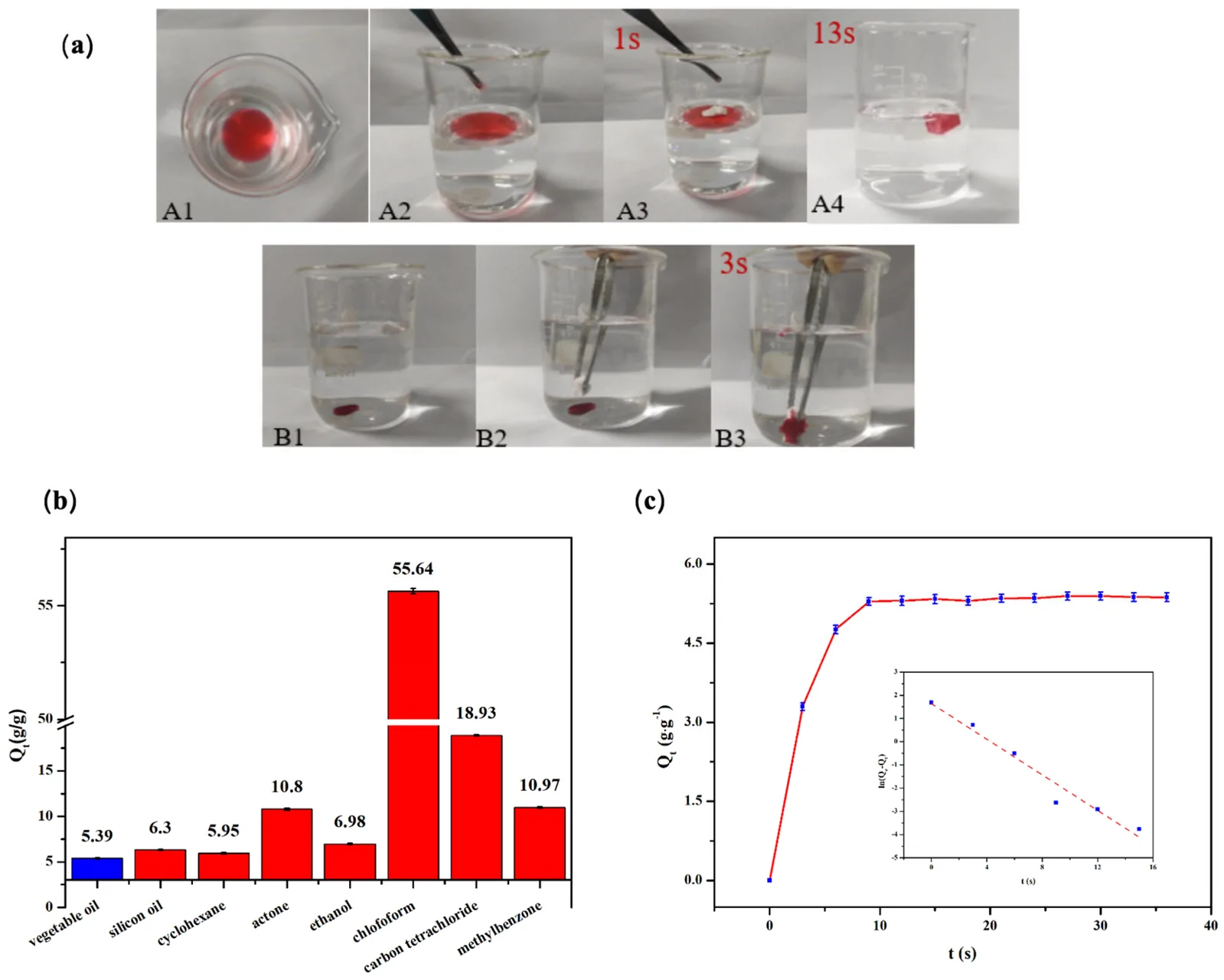
(**a**) Photographs of the separation processes of vegetable oil (A1–A4) and CCl_4_ (B1–B3) from water by using sample I-2; (**b**) saturation absorption capacities of sample I-2 for various oils and organic solvents; (**c**) absorption capacity vs. sorption time and pseudo-first-order kinetics model (inset) of vegetable oil absorption for sample I-2.

**Figure 6:**
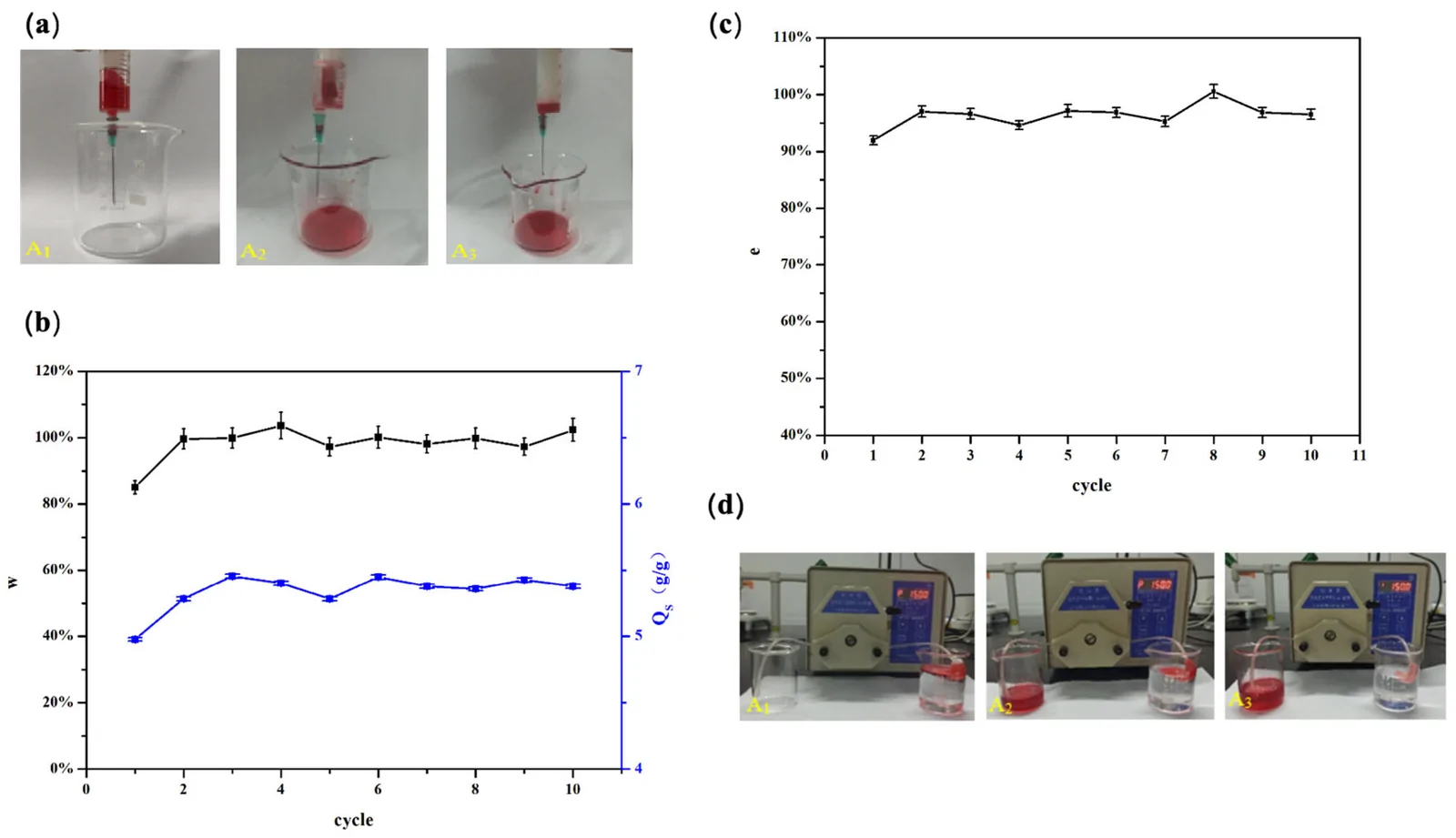
(**a**) Photographs of sample I-2 showing the absorption−squeezing process by hypodermic syringe for removing vegetable oil from water; (**b**) cyclic saturated absorption capacities and recovery efficiency of sample I-2 for vegetable oil; (**c**) cyclic separation efficiency of sample I-2; (**d**) photographs showing the pump-assisted continuous removal of vegetable oil (dyed with Sudan IV) from water (dyed with methyl blue).

**Table 1:** Adsorption efficiency of macroporous TPU gels toward trace toluene in oil-in-water emulsions containing organic pollutants under different conditions.

Time	1 mL/30 °C	2 mL/30 °C	2 mL/60 °C
3 min	1.0%	3.02%	88.6%
2 h	15.4%	37.4%	93.8%
6 h	93.4%	97.0%	97.1%

**Table 2:** Experimental parameters and conditions.

No.	Concentration of TPU (g/mL)	1,4-Dioxane/Distilled Water (*v*/*v*)	Phase Separation Time/min	Phase Separation Temperature/°C
I-1	5%	9/1	30	0
I-2	8%	9/1	30	0
I-3	11%	9/1	30	0
I-4	8%	15/1	30	0
I-5	8%	6.3/1	30	0
I-6	8%	9/1	15	0
I-7	8%	9/1	45	0
I-8	8%	9/1	15	4
I-9	8%	9/1	30	4

## Data Availability

The data presented in this study are openly available in the article.
